# Ubiquitin-Specific Protease 1 Promotes Bladder Cancer Progression by Stabilizing c-MYC

**DOI:** 10.3390/cells13211798

**Published:** 2024-10-30

**Authors:** Xia Zhang, Peng Peng, Li-Wei Bao, An-Qi Zhang, Bo Yu, Tao Li, Jing Lei, Hui-Hui Zhang, Shang-Ze Li

**Affiliations:** 1 Department of Laboratory Medicine, School of Medicine, Hunan Normal University, Changsha 410013, China; zx717@hunnu.edu.cn (X.Z.);; 2 School of Medicine, Chongqing University, Chongqing 400030, China

**Keywords:** bladder cancer, USP1, c-MYC, cell proliferation, deubiquitination

## Abstract

Background: Ubiquitination is an important post-transcriptional modification crucial for maintaining cell homeostasis. As a deubiquitination enzyme, ubiquitin-specific protease 1 (USP1) is associated with tumor progression; however, its role in bladder cancer is unknown. This study aimed to analyze USP1 expression and study its roles in bladder cancer. Methods: The web server GEPIA was used to analyze the USP1 expression. To explore USP1’s function in bladder cancer, we constructed USP1-knockout cell lines in UMUC3 cells. A FLAG-USP1 (WT USP1) plasmid and a plasmid FLAG-USP1 C90S (catalytic–inactive mutant) were used to overexpress USP1 in T24 cells. CCK8, colony formation, and Transwell assays were used to assess cell viability, proliferation, and migration. RNA-sequencing (RNA-seq) and dual-luciferase reporter assays were performed to screen the pathway. Co-immunoprecipitation and immunofluorescence were used to explore the interaction between USP1 and c-MYC. A xenograft mouse model was used to study the role of USP1 in bladder cancer. Results: USP1 expression was upregulated in human bladder cancer cells and correlated with poor patient prognosis. USP1 overexpression promoted cell proliferation, clone formation, and migration, and this was attenuated by genetic ablation of USP1. Furthermore, we observed that *USP1* deficiency inhibited tumor formation in vivo. Mechanistically, the c-MYC pathway was remarkably activated compared with the other pathways. Furthermore, USP1 could interact with c-MYC and increase c-MYC’s stability depending on the catalytic activity of USP1. Conclusions: Our results suggested that high expression of USP1 promotes bladder cancer progression by stabilizing c-MYC; hence, USP1 may serve as a novel therapeutic target for treating bladder cancer.

## 1. Background

Bladder cancer involves a tumor of the genitourinary system and is the sixth most common cancer affecting males globally [1]. Depending on the degree of invasion, bladder cancer can be classified into the following two types: non-muscle-invasive bladder cancer (NMIBC), which is confined to the bladder mucosa and submucosa and accounts for approximately 70–75% of newly diagnosed cases, and muscle-invasive bladder cancer (MIBC), in which tumor cells invade deeper layers of the bladder wall or form metastases, accounting for the remaining 25–30% of cases [2]. The five-year survival rate of patients with NMIBC is approximately 90%; however, the frequency of short-term recurrence after the initial tumor excision is 50–70%, and 10–20% of NMIBC cases may progress to MIBC. The five-year survival rate of MIBC is approximately 60%, which may be reduced to less than 10% in cases of early metastatic dissemination. Unlike many other cancers, systemic therapy and survival rates for bladder cancer have remained largely unchanged over the past three decades [3]. Therefore, improved understanding of the molecular biology of bladder cancer is important for the development of diagnosis and treatment.

Ubiquitination is an essential post-translational protein modification involved in many cellular processes, including protein degradation and interactions, cell survival, apoptosis, and autophagy [4,5]. Disorders of the ubiquitin system may lead to many conditions, such as cancer, neurodegenerative diseases, and infectious diseases [6,7,8]. Deubiquitinating enzymes (DUBs) play a critical role in maintaining the balance of the ubiquitin system by removing ubiquitin from ubiquitin-bound proteins and preventing proteasomal degradation. Therefore, DUBs can regulate the expression and activities of various proteins, including tumor suppressors, and have recently been regarded as novel potential targets for cancer treatment. Ubiquitin-specific protease 1 (USP1) is a well-characterized DUB containing the catalytic residues Cys90, His593, and Asp751, which relate to its deubiquitination activity. USP1 plays an important role in regulating DNA damage repair, mainly via Fanconi anemia pathway activation and trans-lesion DNA synthesis [9,10]. Recent studies have suggested that USP1 contributes to tumorigenesis and the progression of various cancers. Its expression is upregulated in ovarian cancer, gastric cancer, hepatocellular carcinoma, renal clear carcinoma, breast cancer, and diffuse large B-cell lymphoma and is correlated with poor prognosis [11,12,13,14,15,16]. A reduction in USP1 levels inhibits cancer cell proliferation, suppresses metastasis, and sensitizes cancer cells to irradiation and chemotherapeutic drugs in various cancers [17,18]. However, the effect and molecular mechanisms of USP1 in bladder cancer have not been reported.

We found that USP1 overexpression promoted the proliferation and migration of T24 bladder cancer cells. Consistent with these results, USP1 deficiency inhibited the proliferation and migration of UMUC3 bladder cancer cells. Next, we explored the mechanisms and found that USP1 bound to and stabilized c-MYC by mediating its deubiquitination, thereby promoting the progression of bladder cancer. Furthermore, we observed that *USP1* deficiency inhibited tumor formation in vivo. All the results suggested that *USP1* is an oncogene in bladder cancer, which promotes cancer progression by targeting c-MYC. Finally, we revealed a novel mechanism of *USP1* in bladder cancer development and progression.

## 2. Materials and Methods

### 2.1. Cell Lines and Cell Culture

All the cell lines were purchased from the American Type Culture Collection (Manassas, VA, USA). HEK293T and UMUC3 (human bladder cancer cell line) cells were maintained in Dulbecco’s modified Eagle’s medium (Gibco, Thermo Fisher Scientific, Waltham, MA, USA), and T24 cells were maintained in McCoy’s 5A medium (HyClone, Sigma-Aldrich, St. Louis, MO, USA), supplemented with 10% fetal bovine serum (Gibco) and 100 U penicillin/streptomycin (Gibco) at 37 °C in a 5% CO_2_ incubator.

### 2.2. Antibodies

Anti-USP1 (8033) and anti-c-MYC (18583) antibodies were purchased from Cell Signaling Technology (Beverly, MA, USA). Anti-MYC-tag-monoclonal antibody (mAb; M192-3), anti-HA (561), anti-Flag (PM020), and anti-GAPDH (M171-3) antibodies were obtained from MBL (Nagoya, Japan). Rabbit mAb c-MYC (a1309), goat anti-mouse IgG-Cy3 (AS008), and goat anti-rabbit IgG-FITC (AS011) were obtained from ABclonal (Woburn, MA, USA). Rabbit polyclonal antibody (pAb) against USP1 (14346-1-AP) and mouse mAb against GAPDH (60004-1) were purchased from Proteintech (Wuhan, China).

### 2.3. Construction of USP1-Overexpressing and USP1-Knockout Cell Lines

T24 cells overexpressing *USP1* were established via lentiviral infection. In brief, full-length *USP1* and *USP1* catalytic–inactive mutants (C90S) were cloned into the lentiviral vector pHAGE-3 × flag (Addgene, Cambridge, MA, USA). For lentiviral production using the pHAGE vector, packaging psPAX2 and envelope pMD2.G plasmids (Addgene) were co-transfected into HEK293T cells using a transfection reagent. The lentivirus particles were then used to infect T24 cells with 5 mg/mL of polybrene. After 48 h of infection, 0.5 mg/mL of puromycin was added to the medium to select stable clones.

*USP1*-knockout cells were obtained by clustered, regularly interspaced, short palindromic repeats (CRISPR)/Cas9-mediated genome editing [19]. The sgRNAs were designed via the CRISPR Design Tool (sgRNA1: 5′-CTGTTCAACCGAAATGATTA-3′; sgRNA2: 5′-AAGCAGTCGCCTTGGCTGAG-3′). The annealed sgRNA oligos were inserted into the lentiCRISPRv2 vector (Addgene plasmid 52961) [20] to generate the USP1-knockout plasmids. The vector was transfected into HEK293T cells with the pMD2.G and psPAX2 plasmids to produce lentivirus particles. After 48 h of lentiviral infection of UMUC3 cells, 1 mg/mL of puromycin was added to the medium, and the limiting dilution method was used to screen for monoclonal cells.

### 2.4. Western Blot and Co-Immunoprecipitation

Cell lysates were extracted using sodium dodecyl sulphate sample buffer (Beyotime, Shanghai, China), and their concentrations were measured using the BCA assay kit (Beyotime). Equal amounts of protein were separated by 10% SDS-PAGE and transferred to PVDF membranes (Millipore, Billerica, MA, USA). After blocking using 5% skim milk, the membranes were incubated with primary antibodies overnight at 4 °C and secondary antibodies for 1 h at 25 °C. Signals were examined using the Tanon 5500 imaging system (Tanon, Shanghai, China).

For co-immunoprecipitation, cells were lysed using NP-40 lysis buffer (20 mM Tris-HCl (pH 7.4), 150 mM NaCl, and 1% NP-40) with a protease inhibitor cocktail (Beyotime). The supernatant was incubated with the indicated antibodies and protein A/G magarose beads (SM005002, SMART Lifesciences, Changzhou, China) overnight at 4 °C. The next day, the magarose beads were washed with lysis buffer 3 times. The immunoprecipitated proteins were boiled in 2 × SDS-PAGE loading buffer for 10 min at 95 °C. The proteins were detected using Western blotting.

### 2.5. Immunohistochemistry

Bladder cancer tissue microarrays (IWLTN140BL61, Outdo Biotech, Shanghai, China) and xenograft tumor tissue samples were used for immunohistochemistry. The slides were deparaffinized in xylene and rehydrated using an alcohol gradient. After microwave-stimulated antigen retrieval, endogenous peroxidase activity was blocked. After pre-incubation with 10% bovine serum albumin, the slides were incubated with diluted primary and secondary antibodies, and the nuclei were counterstained with hematoxylin. The study was approved by the Ethics Committee of Hunan Normal University.

### 2.6. Cell Proliferation, Colony Formation, and Transwell Assays

A cell-counting kit 8 (CCK-8; BS350B, Biosharp, Hefei, China) kit was used to assess cell proliferation. In brief, a total of 1 × 10^3^ cells/well were seeded in 96-well plates and incubated with the CCK-8 solution for 1 h at 37 °C, following which the absorbance was measured at 450 nm.

For the colony formation assay, 4 × 10^2^ cells were seeded and cultured in 6-well plates for 14 days, and then were treated with paraformaldehyde, followed by staining with 0.025% crystal violet. Images were acquired using a scanner.

For the Transwell assay, cells in serum-free medium were seeded into the upper chambers of Transwell plates (Corning Costar, Corning, NY, USA), complete medium containing 40% fetal bovine serum was placed in the lower chamber, and serum-free medium was added to the upper chamber. Cells migrating to the lower chamber were stained and photographed using an inverted microscope.

### 2.7. Luciferase Assay

A dual-luciferase reporter assay was performed, as previously described [21]. HEK293T cells were transfected with reporter plasmids, pRL-CMV (Addgene), and the *USP1* overexpression plasmid or empty vector. After 48 h, luciferase reporter gene activity was detected using a dual-specific luciferase assay kit (Promega, Madison, WI, USA).

### 2.8. Immunofluorescence

The cells were fixed with 4% paraformaldehyde, and permeabilized with 0.1% Triton X-100. The samples were then stained with primary antibodies and fluorescein-conjugated secondary antibodies. Nuclei staining was performed using DAPI (Beyotime). Finally, the samples were observed and photographed using a confocal laser microscope (Leica SP8, Knowlhill, Milton Keynes, UK).

### 2.9. Xenograft Animal Model

Fifteen female BALB/c nude mice aged six to eight weeks were randomly assigned to the following three groups: UMUC3, control, and USP1^−/−^. Cells were incubated in phosphate-buffered saline at a density of 2.5 × 10^7^ cells/mL. Subsequently, the cells were injected into the dorsal flanks of nude mice (200 µL on each side). When the tumors were visible, the longest and widest tumor lengths were measured using a Vernier scale every other day, and the tumor volume was calculated, as follows: volume = (length × width^2^) × 0.5. Thirty days after inoculation, the mice were deeply anesthetized with isoflurane (5%) for approximately three minutes. Then, the mice were killed by cervical dislocation. The tumors were resected and weighed. All protocols were approved by the Ethics Committee of Hunan Normal University (permit number: 2021286).

### 2.10. Statistical Analyses

Data from three independent experiments were analyzed using GraphPad Prism 8.0 software and shown as the mean ± SEM. Statistical multigroup comparisons were performed by ANOVA, followed by a Student–Newman–Keuls post hoc test. The two-tailed unpaired Student’s *t* test was used in the comparisons of two groups. Correlation analysis was performed by determining Pearson’s correlation coefficient. *p*-Values less than 0.05 were considered statistically significant.

## 3. Results

### 3.1. USP1 Expression Is Upregulated in Bladder Cancer

**Figure 1:**
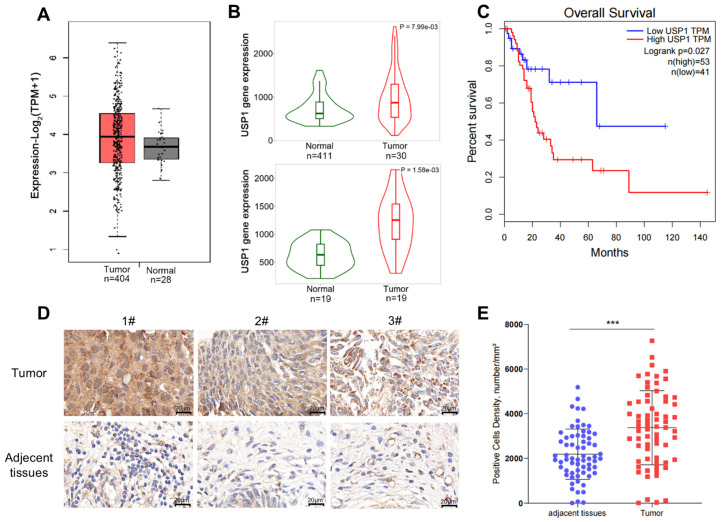
USP1 is overexpressed in bladder cancer. (**A**) USP1 expression in bladder urothelial carcinoma tissues compared with normal tissues in the GEPIA web server. (**B**) USP1 expression in bladder urothelial carcinoma tissues compared with normal tissues in the TNMplot web server (the upper from unpaired tumor and normal tissues, and the lower from paired tumor and normal tissues). (**C**) Analysis of overall survival for 94 patients with bladder cancer based on USP1 expression in the GEPIA web server. The high USP1 group showed significantly lower survival rates (*p* = 0.027). (**D**) Immunohistochemical staining of USP1 in tissue microarrays containing bladder cancer and adjacent tissues. (**E**) Tissue microarray data of USP1 expression in 66 pairs of tumor and adjacent tissues was analyzed by digital pathology image analysis software, and the number of positive cells per square millimeter was calculated (*** *p* < 0.001). Data are presented as the mean ± SD. Statistical significance was analyzed by Student’s *t* test.

We first analyzed the expression of USP1 in multiple types of cancers using the web server GEPIA (http://gepia.cancer-pku.cn/, accessed on 16 May 2024), based on data available from TCGA, and found that *USP1* expression was upregulated in bladder cancer, in comparison with its expression in the normal tissues (Figure 1A). We also performed this analysis using RNA-seq data on the website TNMplot (https://tnmplot.com/analysis/, accessed on 16 May 2024), and the results showed that USP1 expression was significantly increased in tumors. Furthermore, we analyzed USP1 expression in 19 paired tumors and adjacent normal tissues, which showed a significant increase in USP1 expression in tumor tissues (Figure 1B). We also evaluated USP1 expression in bladder cancer using UALCAN (https://ualcan.path.uab.edu/, accessed on 16 May 2024), which indicated that USP1 expression was upregulated in bladder cancer, especially in non-papillary tumors and TP53-mutant tumors; however, no significant differences were observed among the different cancer stages or the nodal metastatic statuses (Figure S1). Next, we analyzed overall survival using the web server GEPIA and found that bladder cancer patients with higher *USP1* expression had poorer survival than those with low *USP1* expression (Figure 1C). We then examined USP1 protein levels in bladder cancer using a tissue microarray with bladder tissues via immunohistochemistry. The results showed that USP1 expression was upregulated in the bladder cancer samples (Figure 1D,E). All the results showed that USP1 is closely related to bladder cancer.

### 3.2. USP1 Overexpression Promotes Cell Proliferation, Migration, and Invasion

**Figure 2:**
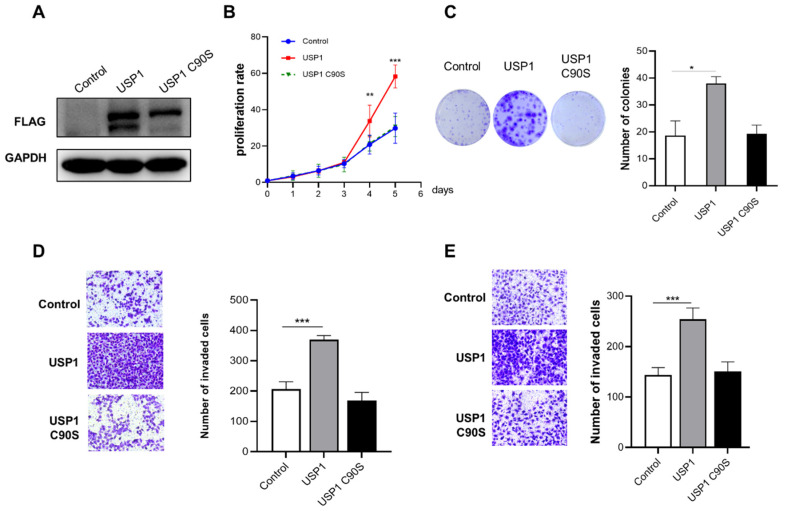
USP1 overexpression promotes bladder cancer cell proliferation, migration, and invasion. (**A**) T24 cells were transfected with FLAG-USP1 or FLAG-USP1 C90S, as indicated, and protein levels were measured by Western blotting. GAPDH served as a control. (**B**) CCK-8 assays were used to analyze cell proliferation (*n* = 6). (**C**) Colony formation assays were performed to evaluate cell viability. The colonies were stained with crystal violet and photographed. The number of colonies was determined and plotted (*n* = 3). (**D**) Transwell experiments were used to evaluate the effects of USP1 overexpression on cell migration. The cells were imaged (left, ×20 magnification) and counted, and the results were plotted (right, *n* = 3). The data (mean ± SEM) are representative of 3 independent experiments. (**E**) Transwell chambers with Matrigel were used to evaluate the effects of USP1 overexpression on cell invasion. The cells were imaged (left, ×20 magnification) and counted, and the results were plotted (right, *n* = 3). The data (mean ± SEM) are representative of 3 independent experiments. Statistical significance was analyzed by ANOVA or Student’s *t* test. * *p* < 0.05, ** *p* < 0.01 and *** *p* < 0.001.

We found that USP1 expression in T24 bladder cancer cells was lower than that in UMUC3 bladder cancer cells. Therefore, T24 cells were selected for transfection with FLAG-USP1 or FLAG-USP1 C90S (a catalytically inactive mutant), and exogenous protein expression was analyzed using Western blotting (Figure 2A). We performed cell proliferation, colony formation, and Transwell assays to determine the effect of USP1 on bladder cancer cells. The results showed that exogenous overexpression of USP1 increased cell proliferation and colony numbers, but overexpression of USP1 C90S did not have the same effects (Figure 2B,C). Transwell assays indicated that the migration and invasion capability of overexpression of USP1, but not USP1 C90S, significantly increased (Figure 2D,E). The above results suggested that USP1 overexpression, but not USP1 C90S overexpression, promotes bladder cancer cell proliferation, migration, and invasion.

### 3.3. USP1-Knockout Represses Cell Proliferation, Migration, and Invasion

**Figure 3:**
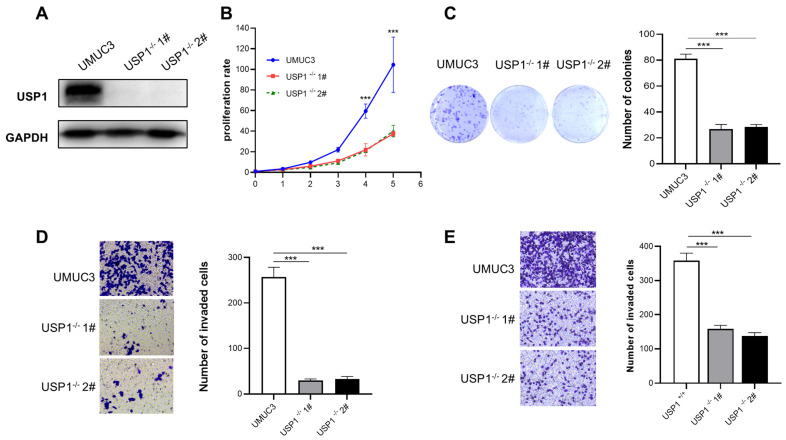
USP1 deficiency inhibits cell proliferation, migration, and invasion. (**A**) USP1 protein levels in WT and USP1-deficient UMUC3 cells were measured by Western blotting, with GAPDH as a loading control. (**B**) Colony formation assays showed the viability of USP1-deficient UMUC3 bladder cancer cells. Colonies were stained with crystal violet and subsequently imaged (left). The number of colonies was determined and plotted (right, *n* = 3). (**C**) Cell proliferation was analyzed by CCK-8 assays with daily measurements for 5 days (*n* = 6). (**D**) Transwell experiments were used to evaluate the effects of USP1 deficiency on UMUC3 bladder cancer cell migration. The cells were imaged (left, ×20 magnification) and counted, and the results were plotted (right, *n* = 3). (**E**) Transwell chambers with Matrigel were used to evaluate the effects of USP1 deficiency on UMUC3 bladder cancer cell invasion. The cells were imaged (left, ×20 magnification) and counted, and the results were plotted (right, *n* = 3). The data (mean ± SD) are representative of 3 independent experiments. Statistical significance was analyzed by ANOVA or Student’s *t* test. *** *p* < 0.001.

The CRISPR/Cas9 system was used to generate UMUC3 *USP1*-knockout cells. Western blotting was used to examine the expression of USP1 (Figure 3A). The results of the CCK-8 assay showed that USP1 deficiency significantly inhibited the proliferation of UMUC3 cells (Figure 3B), and the colony formation capacity of the USP1-deficient cells was lower than that of the wild-type cells (Figure 3C). Moreover, Transwell assays showed that USP1 deficiency repressed the migration and invasion of UMUC3 cells (Figure 3D,E). In summary, USP1 deficiency reduced the proliferation and migration of UMUC3 cells.

### 3.4. USP1 Upregulates the c-MYC Pathway

**Figure 4:**
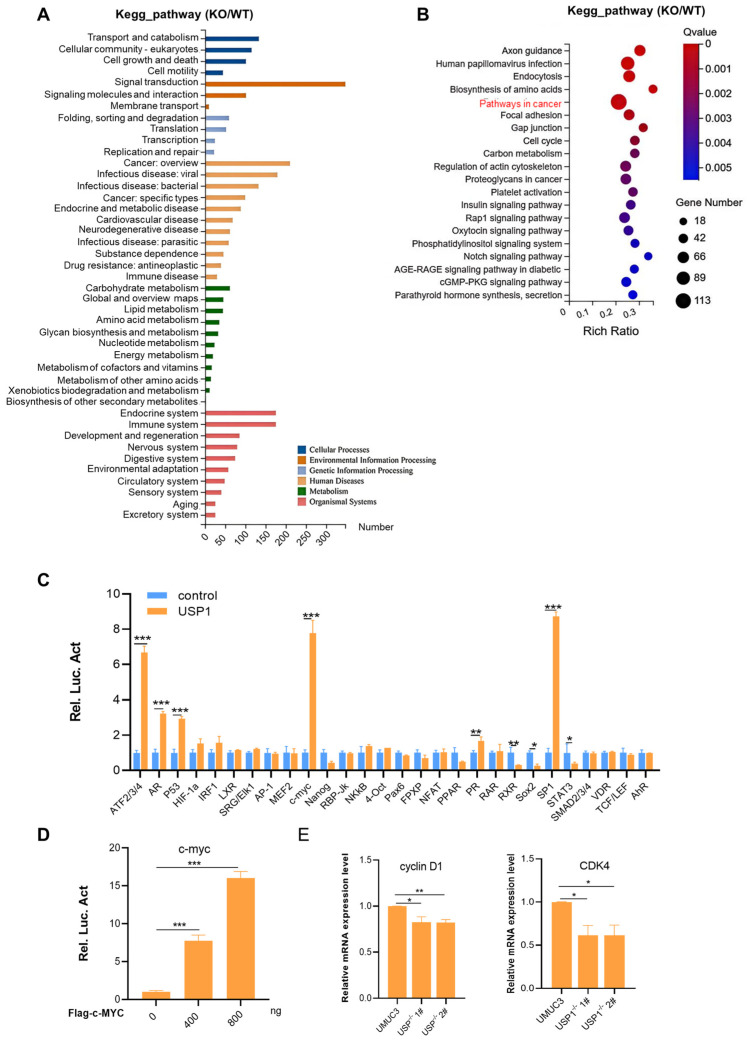
USP1 regulates the c-MYC pathway. (**A**) KEGG pathway enrichment analysis on the basis of the most significantly differentially expressed genes between the WT and USP1-deficient groups (*p* < 0.05 using Fisher’s exact test). (**B**) Bubble diagram showing the enrichment of differentially expressed genes in the biological process category. (**C**) Luciferase pathway screening revealed that USP1 significantly promoted c-MYC pathway activation in HEK293T cells (*n* = 3). (**D**) Luciferase assays showing c-MYC pathway activity in HEK293T cells transfected with increasing amounts (0, 400, and 800 ng) of the USP1 expression plasmid (*n* = 3). (**E**) The transcription of endogenous genes downstream of c-MYC was decreased in USP1-deficient UMUC3 cells, as examined by RT-qPCR (*n* = 3). The data (mean ± SD) are representative of 3 independent experiments. Statistical significance was analyzed by ANOVA or Student’s *t* test. * *p* < 0.05, ** *p* < 0.01, and *** *p* < 0.001.

To explore the mechanism of action of USP1 in bladder cancer cells, we performed RNA-seq analysis using wild-type and *USP1*-knockout UMUC3 cells. The results of the KEGG pathway enrichment analysis indicated that multiple signaling pathways involving numerous biological processes were affected in USP1-deficient UMUC3 cells (Figure 4A). The ratio of total genes enriched by pathways in cancers was the largest (Figure 4B). Next, we used a luciferase reporter gene system to screen for potential pathways in HEK293T cells. The results showed that *USP1* overexpression significantly increased the activity of some signaling pathways, especially c-MYC, ATF2/3/4, and SP1 (Figure 4C). Moreover, USP1 expression increased the c-MYC luciferase reporter activity in a dose-dependent manner (Figure 4D). We examined the expression of downstream target genes of c-MYC, including cycling D and CDK4, in USP1-deficient UMUC3 cells. Compared with parental cells, USP1-deficient UMUC3 cells had decreased mRNA levels of these genes (Figure 4E). The above results showed that USP1 influences bladder cancer cell behavior by regulating c-MYC pathway activity.

### 3.5. USP1 Deubiquitinates and Stabilizes c-MYC

**Figure 5:**
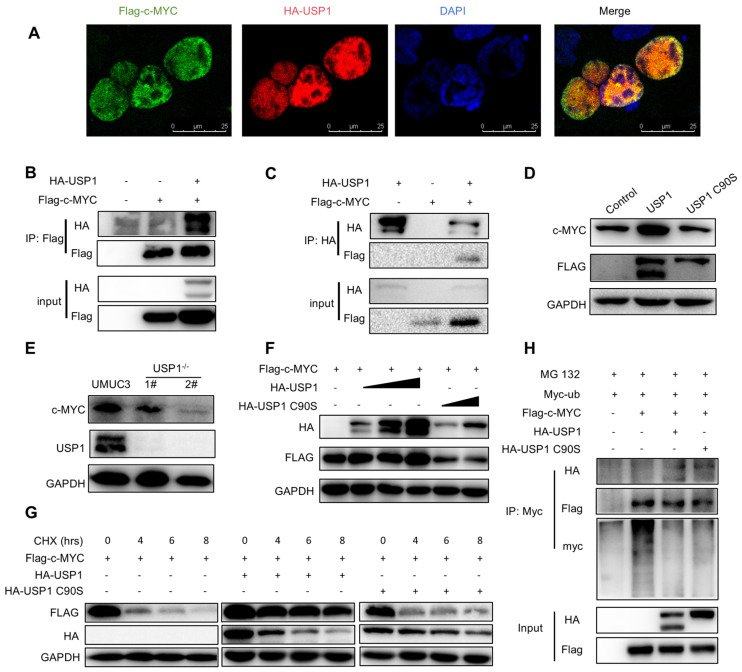
USP1 interacts with c-MYC and promotes c-MYC protein stability. (**A**) Immunofluorescence images of HA-USP1 (red) and FLAG-c-MYC (green) in HEK293T cells. DAPI was used as a nuclear stain (blue). (**B**,**C**) Immunoprecipitation experiments showed the interaction between USP1 and c-MYC in HEK293 cells. (**D**) Western blot analysis of c-MYC expression in USP1-overexpression T24 cells. (**E**) Western blot analysis of c-MYC expression in USP1-deficient UMUC3 cells. (**F**) Cells were transfected with increasing amounts of the HA-USP1 (0, 200, 400, and 800 ng), HA-USP1 C90S (400 and 800 ng), and FLAG-c-MYC plasmids, and Western blotting was performed to determine the effect of USP1 protein levels on c-MYC expression in HEK293T cells. (**G**) Cells were transfected with FLAG-c-MYC with HA-USP1 or HA-USP1 C90S, as indicated. Western blot analysis of c-MYC stability after treatment with CHX (50 μg/mL) for the indicated time. GAPDH served as a control. (**H**) Cells were co-transfected with FLAG-c-MYC and Myc-Ub with or without HA-USP1 or HA-USP1 C90S, treated with MG132 (10 μM) for 6 h, and then subjected to ubiquitination assays.

We next investigated the relationship between USP1 and the c-MYC pathway. We examined the interaction of USP1 with several key proteins. Immunofluorescence staining revealed that USP1 and c-MYC co-localized in the cell nucleus (Figure 5A). To confirm whether USP1 could interact with these proteins, we performed a co-immunoprecipitation assay and found that c-MYC interacted with USP1 (Figure 5B,C). We investigated whether USP1 affects the stability of c-MYC. The results showed that the c-MYC protein level was increased in T24 cells transfected with FLAG-USP1 (Figure 5D), whereas c-MYC levels were decreased in *USP1*-deficient UMUC3 cells (Figure 5E). Since USP1 is a DUB, its function mainly depends on its deubiquitination activity, which may influence the stability of c-MYC. HA-USP1 or HA-USP1 C90S with Flag-c-MYC were transfected to HEK293T cells. The results revealed that USP1, but not USP1 C90S, induced c-MYC expression in a dose-dependent manner, indicating that the DUB activity of USP1 is required for c-MYC stability (Figure 5F). Next, we performed a cycloheximide assay to determine whether c-MYC protein levels are regulated by USP1 over time, and we found that USP1 expression increased the half-life of c-MYC, whereas USP1 C90S failed to rescue the half-life of c-MYC (Figure 5G). Finally, we investigated whether USP1 could inhibit the ubiquitination of c-MYC and found that USP1, but not USP1 C90S, decreased the ubiquitination level of c-MYC, thus increasing its stability (Figure 5H). In summary, these results suggested that USP1 deubiquitinates and stabilizes c-MYC.

### 3.6. USP1-Knockout Inhibits Tumor Formation In Vivo

**Figure 6:**
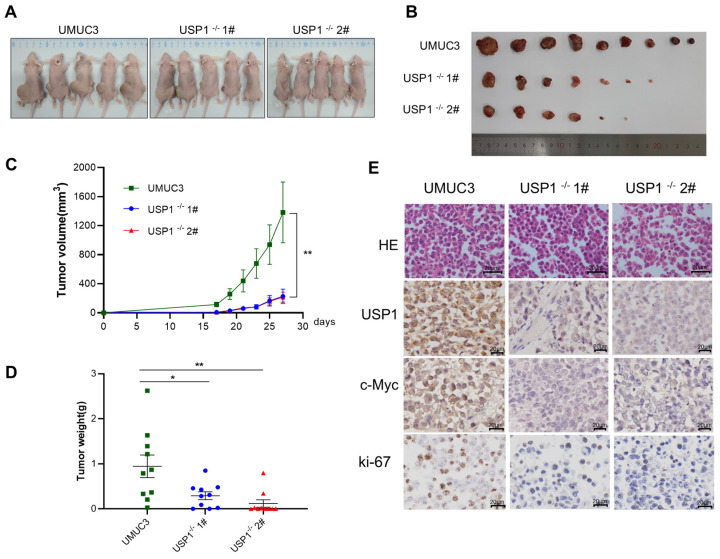
*USP1* deficiency represses tumor formation in vivo. (**A**,**B**) Tumors were harvested from euthanized mice and weighed, and representative images are shown. (**C**) Tumors were measured every 2 days, and the tumor volume was plotted. (**D**) Quantitative results of tumor weight. (**E**) Tumor tissues from each group of nude mice were prepared as paraffin sections and stained with HE, anti-USP1, anti-c-MYC, and anti-Ki67 antibodies. Statistical significance was analyzed by ANOVA or Student’s *t* test. * *p* < 0.05 and ** *p* < 0.01.

To determine whether USP1 can influence tumorigenesis in vivo, we injected WT UMUC3 or *USP1*-deficient UMUC3 cells (USP1^−/−^ UMUC3) into the flanks of BALB/c nude mice. As shown in Figure 6A,B, tumors in mice injected with USP1^−/−^ UMUC3 cells grew markedly slower than those in mice injected with UMUC3 cells, and the tumor volume in nude mice injected with USP1^−/−^ UMUC3 cells was significantly smaller than that in nude mice injected with the WT UMUC3 cells (Figure 6C). Accordingly, the tumor weights of the USP1^−/−^ UMUC3 group were significantly lower than those of the WT UMUC3 group (Figure 6D). Furthermore, immunohistochemical analysis revealed that the expression level of c-MYC was decreased by USP1 deficiency. Additionally, decreased expression of the proliferation marker Ki-67 was observed in the USP1^−/−^ UMUC3 group. (Figure 6E). In summary, our results showed that *USP1* deficiency downregulated c-MYC, which promoted tumor growth in vivo.

## 4. Discussion

USP1 is a deubiquitinating enzyme best characterized for maintaining genome integrity during the DNA damage response. Recently, several studies have indicated that USP1 plays an important role in tumor development; however, the effect of USP1 on bladder cancer remains to be determined. In this study, we analyzed *USP1* expression in the online datasets, which showed that *USP1* was upregulated in bladder cancer. The results were validated by the detection of USP1 expression in immunohistochemically stained tissue microarray samples. Moreover, a high level of *USP1* expression in bladder cancer was correlated with poor prognosis. These results are consistent with those of other studies on ovarian, gastric, and breast cancers [11,12,15].

The silencing or inhibition of *USP1* expression inhibits cell proliferation in osteosarcoma, prostate cancer, and ovarian cancer [22,23,24], but USP1 does not affect the proliferation of tumor cells in liver, gastric, or breast cancers [25,26], indicating that its role differs in various tumors. We used *USP1*-overexpressing T24 and *USP1*-deficient UMUC3 bladder cancer cell lines to ascertain the role of USP1 in bladder tumor development. These two cell lines represent distinct stages of bladder cancer, which may suggest that USP1 may have an effect on different stages of bladder cancer. The phenotype results suggested that USP1 promoted cell proliferation, migration, and invasion. Moreover, we found that *USP1* deficiency inhibited tumor formation in vivo.

Currently, several proteins are deubiquitinated and stabilized by USP1, and this process facilitates cancer progression, including Fanconi anemia group D2 protein and proliferating cell nuclear antigen, which are known to be involved in the DNA damage response pathway [9,10]. Some new substrates of USP1 have also been found in several tumors, such as karyopherin subunit alpha 2 and ERa in breast cancer [15,24], ID2 in gastric cancer [22], c-kit and TBLR1 in liver cancer [23,27], TAZ in osteosarcoma and hepatocellular carcinoma [23,28], Snail in ovarian cancer [18], and PARP1 in cholangiocarcinoma [29]. Here, we performed RNA-seq analysis and luciferase pathway screening to explore the mechanism of USP1 in bladder cancer. The results pointed to the involvement of the c-MYC pathway. We found that USP1 directly interacted with c-MYC.

c-MYC is significantly activated in approximately 70% of human cancers via genetic, epigenetic, and post-translational mechanisms, including the process of maintaining protein stability [30]. The ubiquitin–proteasome system is one of the most prominent protein degradation mechanisms. The deubiquitinating enzyme USP28 can stabilize c-MYC by interacting with FBW7α [31], whereas USP36 deubiquitinates and stabilizes c-MYC by interacting with FBW7γ, but not with FBW7α [32]. To determine whether USP1 stabilizes the c-MYC protein, CHX chase assays and deubiquitination assays were performed, and the results suggested that USP1 stabilized the c-MYC protein by inhibiting its ubiquitination. Consistent with our results, the study in diffuse large B-cell lymphoma (DLBCL) showed that USP1 directly interacted with and stabilized MAX (a MYC-binding protein) through deubiquitination, then promoted the transcription of MYC target genes [16]. However, the binding site that USP1 and c-MYC interact with needs to be further studied.

Regarding the multiple roles of USPs in cellular biological processes, they have been considered candidates for anticancer therapy [33]. USP1 is one of the well-characterized USPs, and its inhibition plays an important role in treating cancers. In DLBCL, inhibition of USP1 expression by shRNA induced apoptosis and autophagy, which caused the reversion of chemotherapy [16]. Human HepG2 cells with USP1 deficiency increased sensitivity to sorafenib by inducing endoplasmic reticulum stress [34]. Recently, the USP1 inhibitor ML323 was shown to inhibit the proliferation of ovarian cancer cells [11] and exhibit anticancer activity against esophageal squamous cell carcinoma [35]. The c-MYC protein is regarded as undruggable because of its largely disordered structure, which lacks a binding pocket for other compounds. Instead of directly targeting c-MYC, modulating the expression of genes interacting with c-MYC may represent a novel strategy in cancer treatment [36]. Our research suggested that USP1 may be a potential target for bladder cancer treatment. Further studies are required to determine whether treatment with USP1-specific inhibitors can inhibit bladder cancer development and progression.

## 5. Conclusions

In summary, we found that *USP1* expression was upregulated and associated with a poor prognosis in bladder cancer. *USP1* is an oncogene that deubiquitinates and stabilizes c-MYC, thereby promoting cancer progression in vitro and in vivo. Our study revealed a new carcinogenic mechanism of action of USP1, which may be a potential therapeutic target for bladder cancer.

## Data Availability

The datasets generated and/or analyzed in this study will be made available by the corresponding author upon reasonable request. The data for RNA-sequencing are available at: http://www.ncbi.nlm.nih.gov/bioproject/970255 (accessed on 9 May 2023).

## Supplementary Materials

The following supporting information can be downloaded at: https://www.mdpi.com/article/10.3390/cells13211798/s1, Figure S1: The analysis of USP1 expression based on tumor histology (A), TP53-mutant status (B), nodal metastatic status (C), and individual cancer stages (D) in bladder cancer. Figure S2: USP1 protein levels in T24 and UMUC3 cells were measured by western blotting with GAPDH as a loading control.
