# Supplementary material for: Ubiquitin-Specific Protease 1 Promotes Bladder Cancer Progression by Stabilizing c-MYC
Source: Cells. 2024 Oct 30;13(21):1798. doi: 10.3390/cells13211798 (PMC11545376; doi:10.3390/cells13211798)
Supplement: Supplementary file 1 [file cells-13-01798-s001.zip › cells-3199489-supplementary.pdf]

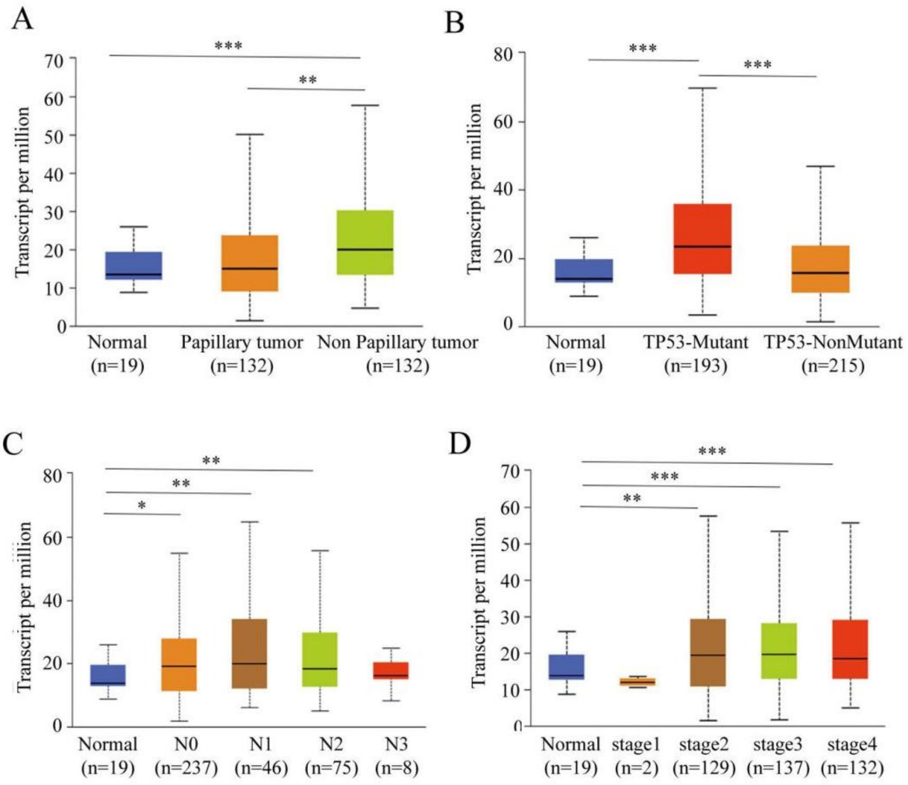

Figure S1 The analysis of USP1 expression based on tumor histology **(A)**, *TP53*-mutant status **(B)**, nodal metastatic status **(C)**, and individual cancer stages **(D)** in bladder cancer.

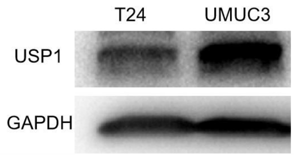

Figure S2 USP1 protein levels in T24 and UMUC3 cells were measured by western blotting with GAPDH as a loading control.
